# Antioxidant activity of *Lactobacillus plantarum* NJAU-01 in an animal model of aging

**DOI:** 10.1186/s12866-021-02248-5

**Published:** 2021-06-15

**Authors:** Qingfeng Ge, Bo Yang, Rui Liu, Donglei Jiang, Hai Yu, Mangang Wu, Wangang Zhang

**Affiliations:** 1 School of Food Science and Engineering, Industrial Engineering Center for Huaiyang Cuisine of Jiangsu Province, Yangzhou University, 225127 Yangzhou, Jiangsu China; 2 Key Lab of Meat Processing and Quality Control, Jiangsu Collaborative Innovation Center of Meat Production and Processing, College of Food Science and Technology, Ministry of Education, Nanjing Agricultural University, 210095 Nanjing, Jiangsu China; 3 College of Food Science and Engineering, Collaborative Innovation Center for Modern Grain Circulation and Safety/Key Laboratory of Grains and Oils Quality Controland Processing, Nanjing University of Finance and Economics, 210023 Nanjing, Jiangsu China

**Keywords:** *Lactobacillus plantarum* NJAU-01, Jinhua Ham, D-galactose-induced aging animal, Malondialdehyde, Antioxidant capacity

## Abstract

**Background:**

Excessive reactive oxygen species (ROS) can cause serious damage to the human body and may cause various chronic diseases. Studies have found that lactic acid bacteria (LAB) have antioxidant and anti-aging effects, and are important resources for the development of microbial antioxidants. This paper was to explore the potential role of an antioxidant strain, *Lactobacillus plantarum* NJAU-01 screened from traditional dry-cured meat product Jinhua Ham in regulating D-galactose-induced subacute senescence of mice. A total of 48 specific pathogen free Kun Ming mice (SPF KM mice) were randomly allocated into 6 groups: control group with sterile saline injection, aging group with subcutaneously injection of D-galactose, treatments groups with injection of D-galactose and intragastric administration of 10^7^, 10^8^, and 10^9^ CFU/mL *L. plantarum* NJAU-01, and positive control group with injection of D-galactose and intragastric administration of 1 mg/mL Vitamin C.

**Results:**

The results showed that the treatment group of *L. plantarum* NJAU-01 at 10^9^ CFU/mL showed higher total antioxidant capacity (T-AOC) and the antioxidant enzymatic activities of superoxide dismutase (SOD), glutathione peroxidase (GSH-Px), and catalase (CAT) than those of the other groups in serum, heart and liver. In contrast, the content of the oxidative stress marker malondialdehyde (MDA) showed lower levels than the other groups (P < 0.05). The antioxidant capacity was improved with the supplement of the increasing concentration of *L. plantarum* NJAU-01.

**Conclusions:**

Thus, this study demonstrates that *L. plantarum* NJAU-01 can alleviate oxidative stress by increasing the activities of enzymes involved in oxidation resistance and decreasing level of lipid oxidation in mice.

## Background

Oxidation is a process necessarily required for cellular metabolism in the body. The reactive oxygen species (ROS) as free radicals produced by endogenous oxidation-reduction (REDOX) reactions were responsible for the oxidation. However, when the cells receive oxidative stress-induced external stimuli, excessive production of ROS overwhelms the cellular ROS scavenging capacity [1]. Some research data have shown that oxidative stress is related with the lifespan of organisms [2]. Unable to metabolize the remaining ROS can cause serious damage to the human body and may cause various chronic diseases related to aging, such as diabetes, heart disease, high blood lipids, arthritis, neurodegenerative diseases, cardiovascular and cerebrovascular diseases [3]. Normally, the body has a series of enzymes or non-enzymes and repair systems which are involved in antioxidant defense and protect them from oxidative damage [4–6]. For example, ascorbic acid consumes oxygen through self-oxidation, reducing metal ions to lower the oxidation-reduction potential and being involved in antioxidant defense [7]. Superoxide dismutase (SOD) is able to convert harmful superoxide radicals into hydrogen peroxide [8]. Catalase (CAT) participates in cellular antioxidant defense by decomposing hydrogen peroxide, thereby preventing the Fenton reaction from producing hydroxyl free radicals [9]. However, superfluous ROS would lead to oxidative damage caused by many factors like irradiation (X-rays, γ-rays, ultraviolet), chemical reagents (metal ions, HONOO, HOCl, and HOBr), drug and their metabolites, and even smoking. These natural antioxidant systems in the body are often insufficient to prevent oxidative damage, requiring antioxidants supplements such as astaxanthin and folic acid [10]. Thus, the search for available approach that can alleviate or inhibit cellular oxidative damage has received considerable attention.

Lactic acid bacteria (LAB) have been widely found and utilized in fermented meat products. Besides improving the nutrient, flavor, and preservation of fermented food, LAB also have additional probiotic characteristics [11, 12]. Some studies have found that LAB have antioxidant and anti-aging effects, and are important resources for the development of microbial antioxidants [13]. For instance, *Lactobacillus case* separated from Chinese homemade liqueur, was found to effectively alleviate lipid peroxidation and improve lipid metabolism due to high cholesterol scavenging ability and human intestinal cell adhesion ability [14]. We have previously isolated and screened a lactic acid bacteria by total antioxidant capacity from Jinhua ham and identified it as *Lactobacillus plantarum* by detecting biochemical characteristics, colonial morphology, and 16 s rDNA sequencing, named *L. plantarum* NJAU-01 [15]. *L. plantarum* NJAU-01 showed excellent scavenging power and reducing power of DPPH free radicals, hydroxyl free radicals, and superoxide anion free radicals *in vitro* [16]. In the cell model coupled with electrochemical sensor, the ability of macrophage RAW264.7 in response to oxidative stress was significantly enhanced by incubation with *L. plantarum* NJAU-01 [15]. In addition, *L. plantarum* NJAU-01 could also alleviate the degree of protein oxidation in fermented sausage [17]. Thus, *L. plantarum* NJAU-01 has been proved to have an antioxidant effect in *vitro*, which is promising to be potentially utilized in regulating oxidative stress in *vivo*.

Thus it is essential to assess the antioxidant effect of *L. plantarum* NJAU-01 *in vivo* by using the animal model, which can effectively observe the absorption, transportation and metabolism in animals. D-galactose-induced aging mice have been developed to simulate the occurrence of oxidative damage in the aging process of the body for decades [18]. D-galactose contributes to the generation of ROS via reaction with amino acids to form glycation end products through non-enzymatic glycation with the benefits of low toxicity, slow oxidation process, and no lethal effect [19, 20]. D-galactose-induced aging mice model has often been reported to evaluate the antioxidant capacity of probiotics such as L-carnitine, ursolic acid and *L. plantarum* AR501 [19, 21, 22]. Therefore, this mature D-galactose-induced aging mice were used for preliminary investigation of the role of *L. plantarum* NJAU-01 in relieving oxidative stress in mice. *L. plantarum* NJAU-01 was fed to D-galactose-induced aging mice to evaluate its *in vivo* antioxidant effects by measuring the total antioxidant capacity (T-AOC), the antioxidant enzymatic activities of SOD, glutathione peroxidase (GSH-Px), and CAT, as well as the content of the oxidative stress marker malondialdehyde (MDA) in mice serum, heart and liver. This will provide the basis for further research on the development and utilization of *L. plantarum* NJAU-01 as a probiotic.

## Results

### Body weight and organ indices in mice

None of the animals died during the feeding period and date from all mice were included. The effects of different treatments with *L. plantarum* NJAU-01 on the organ indices of mice are shown in Table 1. The mice in aging group injected with D-galactose showed significantly lower body weight compared with that of the other groups (*P* < 0.05). Also, no significant difference was observed in kidney index, liver index and lung index between the *L. plantarum* NJAU-01 treatment groups and the normal group, the positive group and the aging model group (*P* > 0.05). The heart index of the aging model group was significantly higher compared to that of the normal group (*P* < 0.05).

**Table 1:** The body weight and organ coefficient in mice among different treatments

Group	Body weight (g)	Organ index (%)				
		Heart	Kidney	Spleen	Liver	Lung
CK	28.28 ± 0.25^a^	0.41 ± 0.04^b^	1.02 ± 0.14^a^	0.22 ± 0.04^b^	3.83 ± 0.44^a^	0.56 ± 0.07^a^
Vc	28.85 ± 0.63^a^	0.46 ± 0.05^ab^	1.02 ± 0.11^a^	0.28 ± 0.08^a^	3.66 ± 0.53^a^	0.60 ± 0.09^a^
SL	25.60 ± 0.34^b^	0.48 ± 0.07^a^	1.07 ± 0.17^a^	0.26 ± 0.04^ab^	4.01 ± 0.58^a^	0.58 ± 0.11^a^
LP1	27.58 ± 0.42^a^	0.41 ± 0.04^b^	1.04 ± 0.13^a^	0.21 ± 0.04^b^	3.71 ± 0.68^a^	0.55 ± 0.06^a^
LP2	28.60 ± 1.51^a^	0.41 ± 0.05^b^	1.02 ± 0.10^a^	0.23 ± 0.05^ab^	3.69 ± 0.61^a^	0.57 ± 0.10^a^
LP3	29.28 ± 2.12^a^	0.46 ± 0.06^ab^	1.03 ± 0.14^a^	0.26 ± 0.03^ab^	3.65 ± 0.80^a^	0.62 ± 0.06^a^

Note: The data presented as the means ± SD (n = 8). Organ indices = weight of organ/body weight ×100. Different letter in the column ^a,b^ shows significant difference among treatments at *P* < 0.05. SL indicates mice group injected with D-galactose. CK indicates injection of sterile saline (0.85 % NaCl) into mice instead of D-galactose. Supplement of Vitamin C after D-galactose injection is named as Vc group. The *L. plantarum* NJAU-01 was grown in De Man, Rogosa, Sharpe (MRS) at 37 °C for 18 h. The intact cells were washed by sterile saline for three times and then adjusted to 1.0 × 10^9^ CFU/mL, 10^8^ CFU/mL, and 10^7^ CFU/mL, respectively. Supplement of *L. plantarum* NJAU-01 at 10^7^ CFU/mL, 10^8^ CFU/mL, and 10^9^ CFU/mL level after D-galactose injection are designated as LP1, LP2 and LP3, respectively

### T-AOC activity of serum, heart and liver of mice

**Fig. 1:**
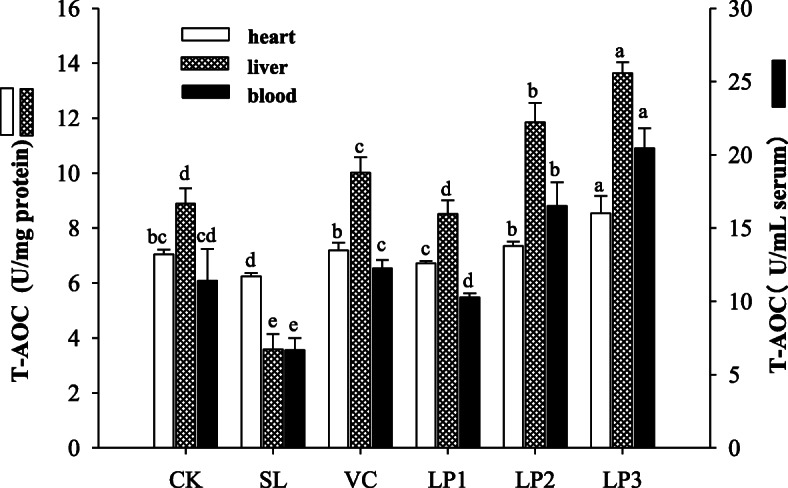
T-AOC activity of blood, heart and liver of mice in different groups. Different lowercase letters means significant difference in T-AOC activity between the same organ of different treatment groups (*P* < 0.05). SL indicates mice group injected with D-galactose. CK indicates injection of sterile saline into mice instead of D-galactose. Supplement of Vitamin C after D-galactose injection is named as Vc group. Supplement of *L. plantarum NJAU-01* at 10^7^ CFU/mL, 10^8^ CFU/mL, and 10^9^ CFU/mL level after D-galactose injection are designated as LP1, LP2 and LP3, respectively. The same abbreviations were used in following figures

The T-AOC activity of mice among different treatment groups is shown in Fig. 1. The T-AOC activity of the aging mice model group was 6.68 U/mL, and while in heart, serum and liver were 6.62, 3.55 and 3.58 U/mg protein, respectively, which were lower than other groups (*P* < 0.05). In the *L. plantarum* NJAU-01 LP3 group, serum, heart, and liver had significantly higher T-AOC than the other groups (*P* < 0.05). The liver T-AOC in the positive control group was significantly higher than the control group and the aging model group (*P* < 0.05). However, the antioxidant activity of the heart and liver of the positive control group was significantly lower than that of the LP3 group (*P* < 0.05). The above results indicate that *L. plantarum* NJAU-01 can enhance the T-AOC of the mice in a dose-dependent manner.

### SOD activity in mice serum, heart and liver

**Fig. 2:**
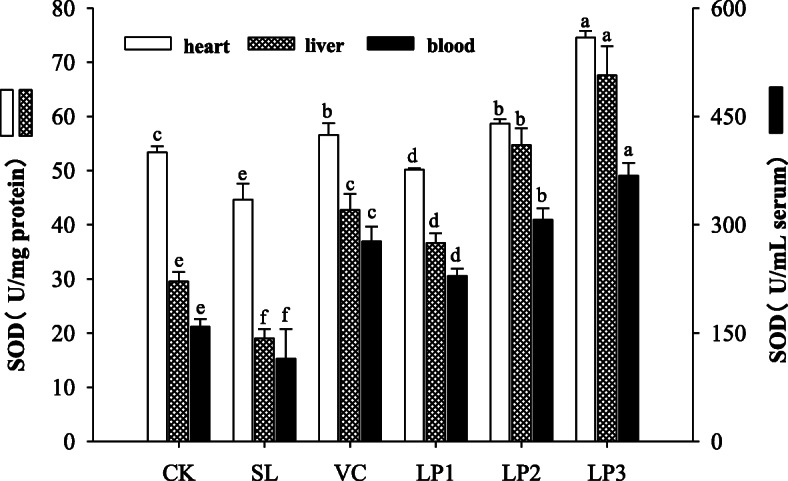
SOD activity of blood, heart and liver of mice in different treatment groups. Different lowercase letters means significant difference in SOD activity between the same organ among treatment groups (*P* < 0.05)

The SOD activities of mice in the aging model group in serum, heart and liver of mice were 16.72 U/mL, 18.93 U/mg protein and 44.82 U/mg protein respectively, which were significantly lower than the control group (*P* < 0.05, Fig. 2). In contrast, the SOD activity in serum, heart and liver of mice in Vc group was significantly higher than that of mice in the aging model group and control group (*P* < 0.05). The SOD activity in the heart of mice in LP2 group was 61.85 U/mg protein, which was not significantly different from that in the positive control group (*P* > 0.05). On the other hand, the SOD activities in the serum, heart and liver of high dose group were 48.83 U/mL, 74.67 U/mg protein and 69.55 U/mg protein respectively, which were significantly higher than that in the other groups (*P* < 0.05). These results showed that *L. plantarum* NJAU-01 could alleviate the oxidative damage induced by D-galactose to the body, and increase SOD activity.

### GSH-Px in mice serum, heart and liver

**Fig. 3:**
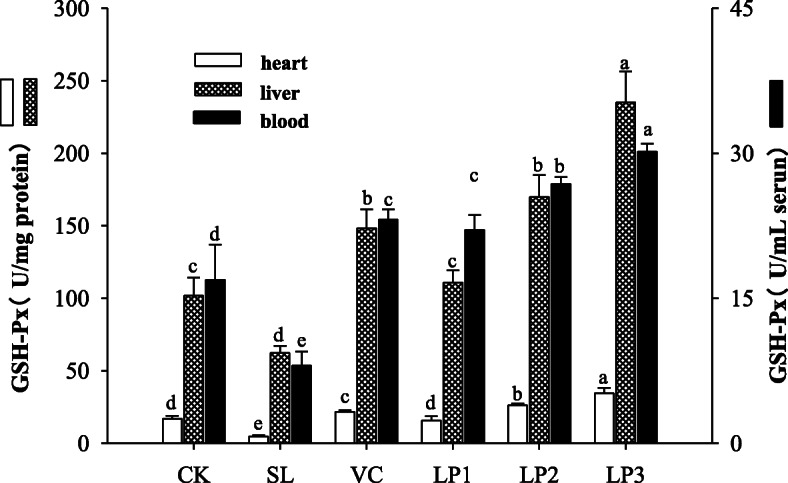
GSH-Px activity in blood, heart and liver of mice among treatment groups. Different lowercase letters means significant difference in GSH-Px activity between the same organ of different treatment groups (*P* < 0.05)

LP3 group presented higher GSH-PX activity than that of mice in other groups in serum, heart and liver (*P* < 0.05, Fig. 3). The GSH-Px activity in the heart and liver tissues of mice in the positive control group was significantly higher than that of mice in control group and the aging model group (*P* < 0.05). There was no significant difference for the serum GSH-Px activity between the LP1 and control group (*P* > 0.05). In addition, the aging model groups in the heart and liver of mice were 50.39 U/mL, 8.48 U/mg protein and 62.67 U/mg protein respectively, showing lower GSH-Px activity than the other groups (*P* < 0.05). Therefore, *L. plantarum* NJAU-01 has an enhancing effect on the antioxidant enzymatic activity of GSH-Px in mice, and the strain concentration is related to the antioxidant effect.

### CAT activity in mice serum, heart and liver

**Fig. 4:**
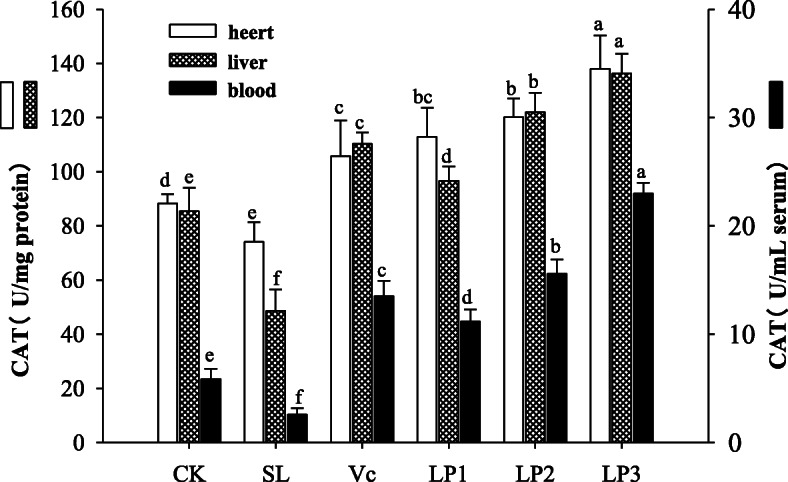
CAT activity of blood, heart and liver of mice in different groups. Different lowercase letters means significant difference in CAT activity at the same parts of mice among different treatment groups (*P* < 0.05)

As shown in Fig. 4, the CAT activities of serum, heart and liver of LP3 mice were 22.98 U/mL, 137.99 U/mg protein and 136.31 U/mg protein respectively, which possessed higher CAT activity than that in other treatment groups (*P* < 0.05), indicating that the concentration of the strain had a marked effect on the CAT activity. In contrast, the aging model group showed lower CAT activity than that of mice in the other groups in serum, heart and liver of mice (*P* < 0.05). As for Vc group, it presented higher CAT activity than the control group and aging model group in serum, heart and liver of mice (*P* < 0.05). These findings demonstrate that *L. plantarum* NJAU-01 can enhance the CAT activity in mice serum, heart and liver tissues.

### MDA content in mice serum, heart and liver

**Fig. 5:**
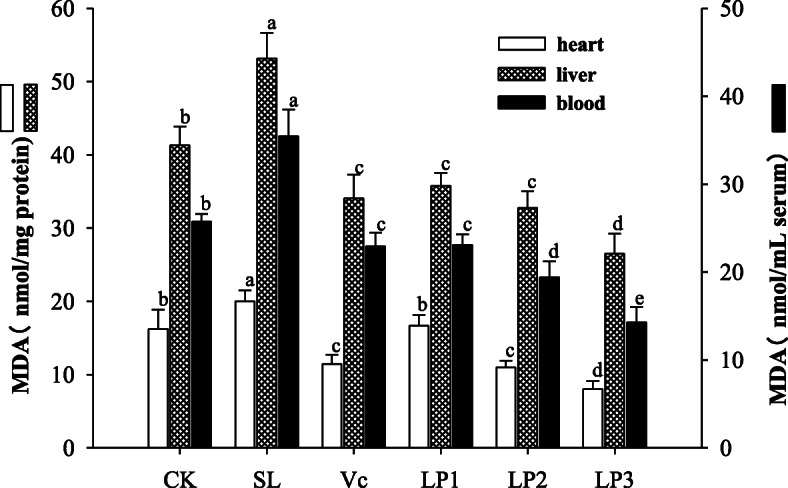
The MDA content of blood, heart and liver in different treatment groups. Different lowercase letters in the figure show significant differences in MDA content among different groups (*P* < 0.05)

MDA content in serum, heart and liver of mice in the aging model group was significantly higher than the control group (*P* < 0.05, Fig. 5). The serum MDA content of mice in the LP3 group was 14.29 nmol/mL, and the MDA contents in the heart and liver were 8.00 and 26.49 nmol/mg protein, respectively, being significantly lower than that in the other groups (*P* < 0.05). There was no significant difference in MDA content among the positive control group, LP1 and LP2 groups (*P* > 0.05).

## Discussion

The subacute aging mice model by the D-galactose induction is widely used and well-recognized [14, 23, 24]. The model involves continuous injection of D-galactose which is reduced to galactose by galactose reductase inside the cells, resulting in change of osmotic pressure between the cell and the environment and then cell swelling and aging [25]. Intracellular enzymes that relieve oxidative stress, such as SOD, CAT and GSH-Px, are not sufficient to eliminate reactive oxygen species, when the cells in the body are subjected to acute oxidative stress. It is a direct and effective method to study the antioxidant activity of LAB by injecting LAB into D-galactose-induced subacute aging mice and comparing enzymatic antioxidant activity, such as SOD, GSH-Px, and CAT with the control group [21].

In this study, the aging model group and the positive control (injection with Vc) group were compared with different doses of the *L. plantarum* NJAU-01 treatment group. The organ index is the ratio of the weight of an organ in the experimental animal to its body weight and an increase in the organ coefficient indicates congestion, edema, or hyperplasia of the organ while a decrease in the organ coefficient indicates organ atrophy and other degenerative changes. The organ index was also used to express the changes in degree of aging as evidenced in studies of Yu et al. (2016) and Xu et al. (2016) [18, 26]. The present study revealed that the mice in aging model group had lower weight and higher heart index than that of mice in the other groups. The administration of D-galactose significantly decreased their antioxidant enzymatic activity in serum, heart and liver of mice in the aging model group. The *L. plantarum* NJAU-01 supplements was suggested to reduce the liver injury of D-galactose-induced oxidative stress mice by regulating abnormal activities of SOD, GSH-Px, and CAT to normal levels. It is in accord with the reported studies when investigating the antioxidant role the targeted strains including *L. plantarum AR113* and *AR501* [12], *L. delbrueckii subsp. bulgaricus* F17 [27]. Thus, D-galactose subacute mice model of aging in the current study is efficient to provide the confidential evidence for investigating the antioxidant capacity of LAB *in vivo*.

Previous research results have indicated that the antioxidant mechanism of LAB is mainly manifested in the following aspects: chelated metal ions, autotrophic antioxidant enzyme systems, production of antioxidant metabolites, increased host antioxidant enzyme activity, control of antioxidant signaling pathways, and regulation of intestinal bacteria group [28]. These aspects play a critical role in alleviating diseases whose development involves with oxidative stress [29]. MDA is considered as the biomarker of lipid peroxidation, and it can lead to cross-linked polymerization of macromolecules, playing a potential role in cytotoxicity and genotoxicity. The content of MDA is usually used as a basis for evaluating the degree of lipid peroxidation and reflecting the level of damage to cells [30]. The current study found that mice with D-galactose-induced oxidative stress had significantly reduced MDA levels in serum, heart and liver by injecting with *L. plantarum* NJAU-01 indicating that *L. plantarum* may effectively reduce the formation of lipid peroxide in mice. This is consistent with *in vitro* study, which showed effective free radical scavenging ability of the *L. plantarum NJAU-01* strain [16]. Similarly, weaned piglets fed with *L. plantarum* ZLP001 presented a lower serum MDA content (4.1 nmol/ml) than the control group (6.23 nmol/ml), demonstrating that *L. plantarum* ZLP001 possessed antioxidant activity [31]. The current study suggested that *L. plantarum* NJAU-01 supplements could essentially relieve the degree of lipid oxidation in D-galactose-induced mice, protecting the mice from oxidative stress.

There is a set of free radical scavenging enzyme defense systems in the body, such as SOD, GSH-Px and CAT, which synergistically scavenge superoxide radicals, hydroxyl radicals and hydrogen peroxide, respectively [32]. The free radical oxidation and the antioxidant defense systems of the organism are in a state of dynamic equilibrium. When the body is exposed to oxidative stress inducing stimuli, this dynamic balance might be disrupted. Excessive production of ROS damages proteins, lipids and nucleic acid molecules, ultimately leading to the aging of organisms and the development of various diseases [33]. SOD can convert superoxide radicals to hydrogen peroxide, which is still cytotoxic and can generate hydroxyl radicals by Fenton reaction [34]. Hydroxyl radical is one of the most active ROS, which can react with organic matter in the cell with the fast reaction rate and destructive effect [35]. Moreover, CAT can decompose hydroxyl radicals to participate in cell antioxidant defense [36]. Under physiological conditions, antioxidant enzymes, such as GSH-Px can be produced in cells to protect these cells from oxidative damage [25]. This study showed that *L. plantarum * NJAU-01 can significantly enhance the activities of SOD, GSH-Px, CAT and T-AOC in serum, heart and liver of mice, indicating that *L. plantarum* NJAU-01 alleviates the oxidative damage caused by D-galactose. This effect may be attributed to two aspects. *L. plantarum* NAJU-01 can promote the activity of antioxidant enzymes in mice, regulating the equilibrium of ROS to normal levels in mice. On the other hand, it also could scavenge free radicals and act synergistically with SOD, GSH-Px and CAT to reduce oxidative stress. Regulation of activities of antioxidant enzymes by the probiotic bacteria was also reported for *Lactobacillus fermentum* [37] and *Lactobacillus fermentum* ME-3 [38]. This study demonstrates that *L. plantarum *NJAU-01 exerts an antioxidant effect in mice and is a promising alternative to synthetic or plant-derived antioxidants. It is usually used as a bio-source antioxidant for the study of sausage starters or functional products [18]. Although research on the antioxidant activity of LAB has achieved wide attention in recent years, investigations on the underlying mechanism of *L. plantarum* NJAU-01 anti-oxidation, in particular, the metabolic pathways, the protein expression and the regulation of intestinal flora are still scant. Moreover, the cross and complementation of multiple antioxidant mechanisms in lactic acid bacteria requires further investigation.

## Conclusions

This preliminary study evidenced the effect of *L. plantarum NJAU-01* isolated from Jinhua ham on D-galactose-induced aging model of mice. It was found that the addition of *L. plantarum *NJAU-01 during the feeding process of mice can significantly increase the activity of antioxidant enzymes and decrease the content of MDA. This study confirms the possibility of *L. plantarum* NJAU-01 as a bio-antioxidant and lays the foundation for further study of the antioxidant mechanism of *L. plantarum*.

## Methods

### Bacterial strain and animal preparation

*L.plantarum *NJAU-01 (CGMCC14194) was screened from the traditionally dry-cured meat product Jinhua ham using morphological, biochemical and molecular genetic identification methods [15]. This strain had high antioxidant activity and was preserved in the College of Food Science and Engineering of Yangzhou University [16]. *Lactobacillus plantarum* NJAU-01 was preserved as frozen (-80 °C) stocks in De Man, Rogosa, Sharpe (MRS) broth (Bio-way technology Co., Ltd, Shanghai, China) supplemented with 20 % (v/v) glycerol. The strain of 1 % inoculum was activated twice and grown in 10 mL MRS broth at 37 °C for 18 h. The bacterial suspension of 100 µL was then inoculated into the solid MRS medium by the automatic diluter and plater (Reference 414,000, Interscience, Saint-Nom-la-Bretèche, France). The inoculated MRS solid medium was cultured at 37 °C for 24 h, and the viable count was counted by the automatic HD colony counter (Scan 1200, Interscience, Saint-Nom-la-Bretèche, France) and Scan® software version 8.0 (Interscience, Saint-Nom-la-Bretèche, France). The concentration of bacterial suspension was detected to be 2 × 10^9^ CFU/mL, and the strain culture of 10 mL was centrifuged at 6,000 g for 10 min at 4 ℃ to discard the supernatant. The pellet was washed with sterile saline for three times and then dissolved in 20 mL of sterile saline, obtaining 1 × 10^9^ CFU/mL of *L.plantarum *NJAU-01. Then, an aliquot of 2 mL bacterial suspension at 1 × 10^9^ CFU/mL was removed into a new tube to combine with 18 mL of sterile saline for making dose of 1 × 10^8^ CFU/mL bacteria. Analogically, the concentration of 1 × 10^7^ CFU/mL was made by the dilution of 1 × 10^8^ CFU/mL bacterial suspension. The SPF grade KM mice (female, 4 weeks old, weighing 18–20 g) were chosen for experimental animal. Animal feed and bedding were provided by the Institute of Comparative Medicine of Yangzhou University (Yangzhou, Jiangsu, China). All animal experiments were approved by the Animal Welfare and Ethics Committees of Yangzhou University and complied with the guidelines of the Institutional Administrative Committee and Ethics Committee of Laboratory Animals (IACUC license number: 201811009). The mice were raised at 20 ± 2 °C with a relative humidity of 55 ± 5 %. The rats were randomly fed standard rat diet during a half-day light and dark cycle (light phase from 7:00 am to 7:00 pm). Four mice were raised in a cage, fed with pathogen-free diet and water. All materials, including lids, feeders, bottles, and bedding were autoclaved before use. The mice were acclimated for one week before the establishment of mice aging model.

### Establishment of mice model of aging

The subacute D-galactose-induced mice model of aging was established and referred to Zhao et al. with slight modifications [39]. The galactose administration method was conducted by subcutaneous injection in the neck and back. A total of 48 SPF mice were randomly assigned into 6 groups(8 rats/group)after one-week of acclimation. Random numbers were generated using the standard = RAND() function in Microsoft Excel. Mice in every group, except the control group, were subcutaneously injected with 500 mg D-galactose per kg body weight (Shanghai Blue Season Biological Co., Ltd., Shanghai, China) for 4 weeks once a day (D-galactose solution, 50 g/L). The control group was injected with 10 mL sterile saline per kg body weight. In addition, the three treatment groups were given intragastric doses of *L. plantarum* NJAU-01 (10^7^ CFU/mL, 10^8^ CFU/mL, and 10^9^ CFU/mL) at 20 mL per kg body weight, and designated as LP1 group, LP2 group and LP3 group, respectively. The mice in control group and the aging group were intragastrically feed with sterile saline at 20 mL/kg daily. The mice in positive control group were treated with 1 mg/mL vitamin C (Vc) at 20 mL/kg daily. The entire experiment lasted four weeks.

### Preparation of tissue samples

Mice were euthanized to an unconscious state by intraperitoneal injection of 3 % isoflurane. Cessation of heartbeats and non-responsiveness to noxious stimulus (hind paw pinch) were used as criteria to verify death. The eyeball of the mice was removed, and blood was drawn. Then, the blood was immediately centrifuged at 3,000×g for 10 min at 4℃ to obtain the serum and stored at -20℃ until analyzed. After being euthanized, the mice were executed unconsciously by pulling cervical vertebrae, and liver, heart, spleen, kidney, lung and brain were harvested, and weighed to determine the organ indices. The liver and heart samples were homogenized into 10 % tissue homogenate with 0.9 % NaCl, and the supernatant was collected by centrifugation as above-mentioned.

### Parameter detections

The T-AOC and the antioxidant enzymatic activities of SOD, GSH-Px, and CAT, and the contents of MDA were determined using total antioxidant capacity assay kit (ABTS method, A015-2-1), superoxide dismutase (SOD) assay kit (WST-1 method, A001-1-2), glutathione peroxidase (GSH-PX) assay kit (Colorimetric method, A0060201), catalase (CAT) assay kit (Colorimetric method, A007-2-1) and malondialdehyde (MDA) assay kit (TBA method, A003-2-1), respectively. All kits were purchased from Nanjing Jiancheng Bioengineering Institute Co., Ltd (Nanjing, Jiangsu, China). All samples were tested in triplicate, and the detection procedures were conducted in accordance with the instructions.

### Statistical analysis

The data were analyzed using the Data Processing System 7.05 software (Hangzhou Ruifeng Information Technology Co., Ltd., Hangzhou, China). Different of means was compared by the Duncan’s new complex range method. The statistical significance test was performed at the 0.05 level (*P* < 0.05).

## Data Availability

All data generated or analyzed during this study are included in this published article. These data are available from the corresponding author upon reasonable request.

## Abbreviations

SPF: Specific pathogen Free
CFU: Colony-forming unit
T-AOC: Total antioxidant capacity
SOD: Superoxide dismutase
GSH-PX: Glutathione peroxidase
CAT: Catalase
MDA: Malondialdehyde
ROS: Reactive oxygen species
LAB: Lactic acid bacteria
MRS: De Man, Rogosa, Sharpe
